# Bioimaging of botulinum toxin and hyaluronate hydrogels using zwitterionic near-infrared fluorophores

**DOI:** 10.1186/s40824-017-0102-x

**Published:** 2017-10-10

**Authors:** Ki Su Kim, Yun Seop Kim, Kai Bao, Hideyuki Wada, Hak Soo Choi, Sei Kwang Hahn

**Affiliations:** 1 PHI BIOMED Co., #613, 12 Gangnam-daero 65-gil, Seocho-gu, Seoul, 06612 South Korea; 2 Department of Organic Materials Science and Engineering, College of Engineering, Pusan National University, 2 Busandaehak-ro 63 beon-gil, Geumjeong-gu, Busan, 46241 South Korea; 3 Department of Materials Science and Engineering, Pohang University of Science and Technology (POSTECH), 77 Cheongam-ro, Nam-gu, Pohang, Gyeongbuk 37673 South Korea; 4 Gordon Center for Medical Imaging, Department of Radiology, Massachusetts General Hospital and Harvard Medical School, 149 13th Street, Boston, MA 02129 USA

**Keywords:** Hyaluronate, Botulinum toxin, Zwitterionic fluorophore, Tissue augmentation, Bioimaging

## Abstract

**Background:**

The injection of botulinum toxin (BTX) to reduce facial wrinkles is one of the most frequently performed plastic surgery procedures. The biocompatible hydrogels are injected with BTX for effective tissue augmentation. However, it is difficult to determine the interval of injection for effective tissue augmentation.

**Method:**

BTX and hyaluronate (HA) hydrogels were labeled with zwitterionic (ZW) near-infrared (NIR) fluorophores and visualized for 3 weeks after injection to BALB/c nude mice.

**Results:**

BTX-ZW conjugates and diaminohexane (DAH)-HA-ZW hydrogels were successfully prepared by the conventional EDC/NHS chemistry. Using the NIR fluorescence imaging, we confirmed that approximately 10% of BTX-ZW conjugates and 50% of DAH-HA-ZW hydrogels remained 3 weeks post-injection.

**Conclusion:**

This bioimaging technique using invisible NIR fluorescence light can be exploited for various biomedical applications.

## Background

Botulinum toxin (BTX), a neurotoxic protein derived from the bacterium *Clostridium botulinum*, is most frequently used for removing facial wrinkles [1, 2]. Small quantities of BTX can cause relaxation of overactive muscles and reduce wrinkles by smoothing overlying skin [3]. BTX inhibits acetylcholine release and causes temporary chemical denervation at the neuromuscular junction by cleaving the synaptosomal-associated protein of 25 kDa [SNAP-25] on the internal surface of neuronal membranes, followed by vesicle fusion at the cellular level [4]. After SNAP-25 regenerates over time, BTX effects diminish in the targeted muscles, and neuromuscular signaling and muscle contractility are restored [4].

Biocompatible hydrogels are generally injected with BTX to help fill skin wrinkles and effectively augment tissue volume [5, 6]. Among injectable dermal fillers, hyaluronate (HA) hydrogels have become especially popular for soft tissue augmentation since HA can absorb water to recover the volume of aging tissue [7–10]. HA fillers with a larger particle size and higher molecular weight are generally preferred to extend the duration under the skin, which can be obtained by crosslinking HA [11–13]. The typical dose of BTX is 200 unit in 3 months interval [14]. The use of BTX and dermal fillers, however, threatens healthcare workers and patients due to residue left in the body, and it is a challenge to determine the dosing interval for effective tissue augmentation. Moreover, there have been previously reported the efficacy of Botox through bioimaging techniques [14, 15]. However, there were no studies to confirm the in vivo behavior of BTX.

Here, we firstly investigated in vivo dynamics of BTX and dermal fillers for tissue augmentation using near-infrared (NIR) fluorescence, which penetrates deeply into biological tissues [16]. We previously reported that zwitterionic (ZW) NIR fluorophores have low serum binding, low nonspecific tissue uptake, and rapid elimination from the body through renal filtration [17]. ZW fluorophores also have superior optical properties (i.e., high extinction coefficient and quantum yield) compared to visible dyes, which together improves quantitative bioimaging [18–21]. In this study, we investigate the in vivo dynamics of BTX and HA hydrogels for tissue augmentation by introducing ZW fluorophores in the chain.

## Methods

### Materials

Hyaluronate (HA) was purchased from Lifecore Co. (Chaska, MN). Botulinum toxins (BTX, Meditox, Ochang, Korea) were kindly gifted from Dr. Jeesoo An at the Wellman Center for Photomedicine in Massachusetts General Hospital. 1-Ethyl-3-(3-dimethylaminopropyl) carbodiimide (EDC) was obtained from Tokyo Chemical Industry (Tokyo, Japan). N-hydroxysulfosuccinimide (sulfo-NHS), phosphate buffered saline (PBS), diaminohexane (DAH) and hydroxylbenzotriazole (HOBt) were purchased from Sigma (St. Louis, MO). Dialysis membrane tube was obtained from Thermo Scientific Co. (Waltham, MA).

### Synthesis of Zwitterionic (ZW) NIR fluorophores

ZW NIR fluorophores were prepared as reported previously [18]. In brief, Vilsmeier–Haack reagent was used for the condensation reaction with prepared intermediate indolium salts in anhydrous sodium acetate to prepare indocyanine based chloro-subtitued NIR fluorophore. And then, using microwave synthesis, a bifunctional phenoxypropionic acid linkage was introduced on the meso-chlorine atom to permit conjugation of targeting ligands. The crude product was washed against diethyl ether and precipitated in methanol and diethyl ether (20 mL, 1:4) to give the ZW NIR fluorophores.

### Labeling of ZW fluorophores

HA and BTX were labeled with the amine modified ZW fluorophore, with emission wavelengths of 700 nm and 800 nm, respectively. HA (MW 100 kDa) was dissolved at a concentration of 5 mg/mL in double distilled water. After complete dissolution, ZW fluorophores (1 M ratio of HA) were added to the HA solution, and EDC and sulfo-NHS were added with 4 M ratio of HA to activate the carboxyl groups of HA. The pH of the reaction mixture was maintained at 6.5, and ZW fluorophore (1 M ratio of HA) was added to the solution and stirred overnight. After the reaction was stopped by changing the pH to 7.4, the resulting HA-ZW conjugate was purified with gel permeation chromatography (GPC) measuring the retention time. The mobile phase was PBS at pH 7.4 and the flow rate was 1 mL/min. The detection wavelength was 210 nm. The purified conjugate solution was lyophilized for 3 days. BTX was dissolved in phosphate buffered saline (PBS, Sigma, St. Louis, MO) and the conjugation and purification was performed as described above. The detection wavelength was 280 nm.

### Preparation of DAH-HA-ZW hydrogels

DAH-HA-ZW hydrogel was prepared using the same method described elsewhere [22]. HA-ZW conjugates were dissolved at a concentration of 30 mg/mL in double distilled water; after complete dissolution, DAH (1 M ratio of HA) was added to the HA solution for a cross-linking reaction with the carboxyl groups of HA. EDC and HOBt (1 M ratio of HA), activating the carboxyl groups of HA, were dissolved in water and added to the mixed solution of HA and DAH for DAH-HA hydrogel preparation. The final precursor solution was incubated at 37 °C for 2 h to complete the cross-linking reaction. Prepared DAH-HA-ZW hydrogels were sealed within dialysis membrane tube (MWCO of 7 kDa) and dialyzed against PBS for 3 days to remove the remaining EDC, HOBt, and DAH completely. The degree of modification in DAH-HA conjugates was analyzed by proton nuclear magnetic resonance (^1^H NMR, DRX-400, Bruker, Germany).

### Bioimaging of BTX-ZW conjugates and DAH-HA-ZW hydrogels

BTX-ZW conjugates and DAH-HA-ZW hydrogel with the same fluorescence intensity were intramuscularly and subcutaneously inoculated into the BALB/c nude mice (100 pmol of ZW dye, 50 μL), sequentially. The home-built dual-channel imaging system [21] was used to acquire NIR fluorescent images at 0, 1, 2, and 3 weeks post-injection. Animals were housed in an AAALAC-certified facility and all animal studies were performed under the supervision of BIDMC IACUC in accordance with approved institutional protocol of #057–2014.

## Results and discussion

### Synthesis of BTX-ZW conjugates and DAH-HA-ZW hydrogel

**Fig. 1:**
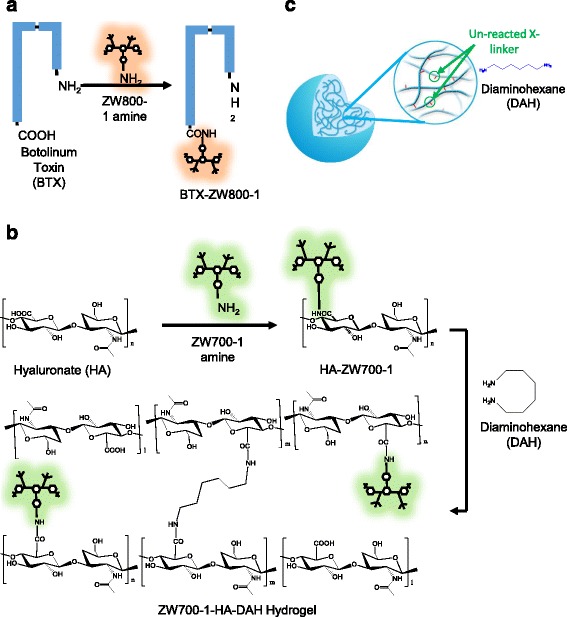
Schematic diagrams for the synthesis of (**a**) BTX-ZW conjugates and (**b**) DAH-HA-ZW hydrogel, and for the illustrated structure of (**c**) DAH-HA hydrogel

Figure 1 shows the schematic representations for the chemical synthesis of BTX conjugates and HA hydrogel with ZW fluorophores. The ZW fluorophores emit NIR wavelength and avoid nonspecific tissue uptake and serum protein association due to their net charge. In plastic surgery, BTXs were typically injected in muscle, which is a deeper site than the subcutaneous injection of hydrogels for tissue augmentation. The carboxyl group of BTX was modified with amine-modified ZW800–1 fluorophore, which has a longer wavelength of fluorescence for intramuscular injection. In addition, HA was conjugated with ZW700–1, which has a shorter wavelength. The ZW labeled HA hydrogel was formed by the crosslinking of DAH. Since the amine groups of unreacted DAH crosslinkers were reported to be nontoxic and promote the attachment and proliferation of keratinocytes and fibroblasts [23], DAH-HA-ZW hydrogel can be exploited for tissue augmentation.

### Characterization of the BTX-ZW, HA-ZW, and DAH-HA conjugates

**Fig. 2:**
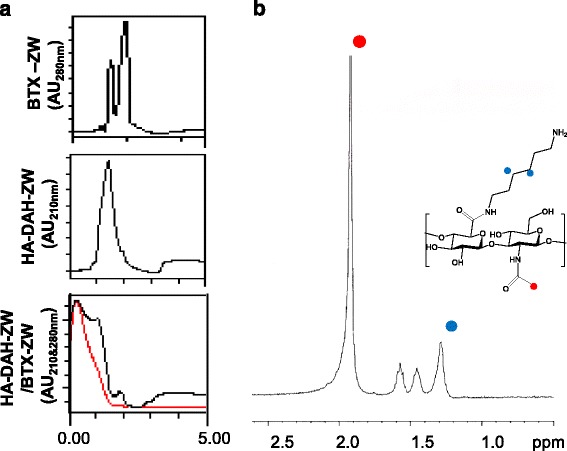
**a** The gel-permeation chromatogram of BTX, HA, and the mixture of HA and BTX. **b** NMR spectrum of DAH-HA conjugates

The resulting BTX-ZW and HA-ZW conjugates were characterized and purified by GPC analysis (Fig. 2a). The retention time of native BTX-ZW with a MW of 150 kDa was ca. 3 min, and the retention time of HA-ZW with a MW of 100 kDa was ca. 1.5 min. The retention time of the mixture of HA-ZW and BTX-ZW was ca. 1.5 min. Successful conjugation of DAH to HA was confirmed by ^1^H NMR analysis (Fig. 2b). The pattern of ^1^H NMR spectrum of DAH-HA conjugates was identical with our previous work [24], and the integral ratio on the ^1^H NMR spectrum suggested that, on average, 18 mol% of HA units was modified with DAH. The methyl resonance (δ = 1.85–2.05 ppm) of acetamido moiety of N-acetyl-D-glucosamine residue in HA was used as an internal standard, and the degree of DAH-HA modification was determined from the peak area of methylenes of DAH at δ = 1.3 ppm.

### Dynamics of BTX-ZW conjugates and DAH-HA-ZW hydrogel

**Fig. 3:**
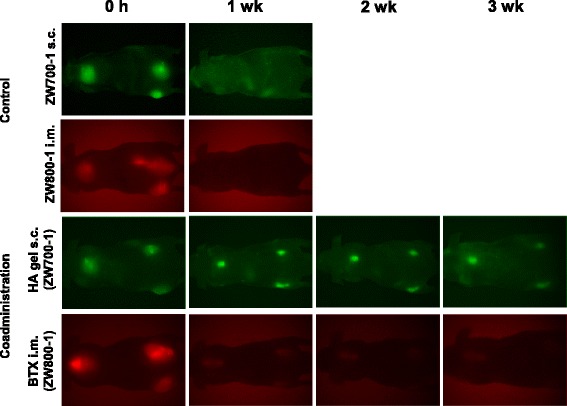
NIR fluorescence images of ZW700–1 with subcutaneous (s.c.) injection, ZW800–1 with intramuscular (i.m.) injection, DAH-HA-ZW hydrogel with s.c. injection, and BTX-ZW conjugates with i.m. injection, in order from top to bottom

Based on characterization, we investigated the dynamics of BTX and HA hydrogel in vivo. The ZW labeled BTX and HA hydrogel were administered with intramuscular and subcutaneous injection, respectively. NIR fluorescent images were acquired with emission wavelength of 700 nm (for ZW700–1) and 800 nm (for ZW800–1) by fluorescence-assisted resection and exploration (FLARE) imaging. As control, ZW800–1 with intramuscular injection and ZW700–1 with subcutaneous injection were also tested for comparison. In Fig. 3, almost no fluorescence signal of control group remained at injection site within 1 week. Based on previous work [20], the control group may also clear within 1 day from the body. BTX-ZW conjugates and DAH-HA-ZW hydrogel remained in the body for over 3 weeks, but the remaining pattern of BTX-ZW conjugates differed from the DAH-HA-ZW hydrogel pattern. The BTX-ZW conjugates cleared quickly, caused by enzymatic degradation. However, since the crosslinked HA can avoid enzymatic degradation, DAH-HA-ZW hydrogel could remain for over 3 weeks.

**Fig. 4:**
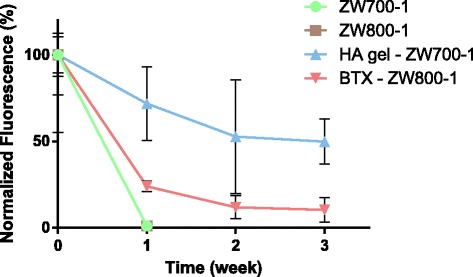
Quantification of fluorescence intensity after i.m injections of ZW800–1 and BTX-ZW conjugates, s.c. injections of ZW700–1 and DAH-HA-ZW hydrogel

Based on FLARE images, the in vivo dynamics of fluorescence was measured by quantifying fluorescence injection of the injection site (Fig. 4). Since all samples with ZW fluorophore were inoculated by injection, we normalized the fluorescence intensity to 100% at 0 week. Both BTX and DHA-HA hydrogel remained in the body longer when compared to the control group. Over 50% HA hydrogel remained subcutaneously, while 10% of BTX remained in muscle after 3 weeks of administration. BTX was easily removed due to its small size and enzymatic degradation. Overall, we could confirm the amount and body residue of BTX and HA hydrogels using bioimaging with ZW fluorophore. This strategy for indicating body residue and dosing interval of agents can be harnessed for various biomedical applications.

## Conclusions

Bioimaging techniques using Zwitterionic (ZW) NIR fluorophores were successfully carried out to investigate in vivo dynamics of each component for tissue augmentation. BTX-ZW conjugates and DAH-HA-ZW hydrogel were synthesized, and in vivo dynamics were investigated using home-built bioimaging equipment. Bioimaging using ZW fluorophores for indication of bodily residue and the interval of agents can be exploited for various biomedical applications.
